# A qualitative study of community perceptions about childhood diarrhea and its management in Assosa District, West Ethiopia

**DOI:** 10.1186/1471-2458-14-975

**Published:** 2014-09-19

**Authors:** Estifanos Yalew

**Affiliations:** Department of Public Health, College of Medicine and Health sciences, Wollo University, Dessie, Ethiopia

## Abstract

**Background:**

Diarrhea control programs require evidences on factors which influence the caregiver’s treatment of illness. Thus, understanding the caregiver’s perception of the causes and management of diarrhea is very essential to plan effective prevention and control measures. This study aimed to explore their perceptions towards the causes and management of childhood diarrhea in Assosa district, West Ethiopia.

**Methods:**

Qualitative research methods were employed among caregivers who reside in two villages (Amba 4 and Selga 22) of the district. The villages were selected purposively and all eligible participants were identified with the help of village leaders and health extension workers. Then, in-depth interviews and focus group discussions were used to collect data from the participants. For this purpose, a semi-structured interview checklist and discussion guides were prepared. Data was collected by experienced and trained sociologists and public health professionals. The collected data was translated and analyzed thematically. No software was used.

**Results:**

Majority of the caregivers perceived inadequate personal hygiene and poor environmental sanitation as the main causes of childhood diarrhea. However, few of them related its occurrence with sucking hot breast milk. On the other side, homemade management of diarrhea was commonly practiced in the community, i.e. providing boiled and cooled water with honey and *Haile Sellasie silver coin [Mariatriza]*. However, indigenous communities preferred traditional medications such as *Sirsafe, Bibi and Kebercho* to their children when they got diarrhea.

**Conclusions:**

Childhood diarrhea was perceived as the commonest disease in the community. Consequently, diverse misperceptions and malpractices on the causes and management of the problem existed. Thus, urgent effective interventions that consider the local culture and resources should be designed.

## Background

Globally, substantial progress has been made towards achieving millennium development goal (MDG 4). As a result, the number of under five deaths worldwide has declined from nearly 12 million in 1990 to 6.9 million in 2011 [1]. Despite the decline, the highest rates of child mortality are still in Sub-Saharan Africa, where one in nine children die before their fifth birthday which is more than 16 times from the average of developed regions [2].

In many developing countries today, especially in Africa, diarrhea still poses a significant threat to the health, wellbeing and survival of under-fives. It also contributes to an enormous share of the disease burden in all age groups, especially children. Of 8.79 million children below the age of five who die worldwide each year, 15% or 1.5 million deaths are due to diarrheal diseases [3].

Ethiopia ranks fifth globally as 20–27% of child deaths are caused by diarrheal diseases [4–6]. The majority of these deaths occur mainly due to fluid loss. This could be easily prevented by caregivers by ensuring early diagnosis and timely administration of oral rehydration therapy, zinc supplementation and continued feeding of an appropriate diet [7]. It follows that evidence on the potential influencing factors is very important to encourage the involvement of families in diarrhea control programs [8]. In addition, understanding the perceptions of caregiver’s on the problem is also very essential for timely and proper management at home and if need be, for skilled care [9]. Therefore, this study aimed to explore the perceptions on the causes and management of childhood diarrhea in the rural district of West Ethiopia.

## Methods

### Study setting

Assosa district is located 676 kilometers from Addis Ababa, in the Western region of Ethiopia. It was selected for the study based on three criteria: (1) Socio-cultural diversity [existence of many ethnic groups such as Berta, Gumuz, Amhara, Shinasha and Oromo], (2) high prevalence of diarrhea, and (3) relatively easy to access given the weather and road conditions. The population is more than 30,000 people and the main source of income is farming and traditional gold mining. The district has about 58 villages with mostly poor infrastructure for electricity, water and health care services. During the time of data collection, two health extension workers were assigned in each village to educate the community about infectious diseases and conduct immunization activities.

### Sample selection

Two villages were selected based on the following criteria: (1) the village provided some ethnic diversity to the sample, (2) the village was outside of a five kilometer radius from the nearest health facility, and (3) the village had access to a passable road. Each village had an approximate population of 1,500 people.

All the study participants were selected purposively and invited to participate in the study. Forty one caregivers and nineteen volunteer community health workers (CHW) were selected. A CHW is a role model mother who has been trained for five days on childhood diseases and is responsible for awareness creation and mobilization of 40 households. She also gives ORS to children who have diarrhea and refer to the nearby health post in case there is no improvement.

Health extension workers (HEW) were also selected. A HEW is a woman who completed 10th or 12th grade and took one year professional training on health extension packages, and serves 1500 people in one village. Three traditional healers, two private institution and two drug vendor owners were also included. All of the selected participants had at least one child less than five years of age.

### Data collection

Semi-structured interview checklists and discussion guides were used for data collection. Data was collected by three experienced sociologists and public health professionals who had received three days training. All research materials were translated into Amharic, the official language of the study area. Upon arrival at the field, a pilot test was conducted with two caregivers and necessary changes were made.

A separate focus group discussions (consisted of 8 to 12 subjects) were conducted with caregivers and volunteer community health workers. Health extension workers, traditional healers, private clinic and drug vendor owners were also approached for in depth interviews.

Mostly probing questions such as ‘What?, ‘Why?’ and ‘How?’ were used during the various sessions. The process continued until a saturation level was achieved (no new information or codes were present). The sessions were conducted at participants’ convenience of time and place.

### Data analysis

All the interviews and discussions were recorded using a tape recorder and transcribed on the same day. The transcribed files were read several times by the research team members and analyzed thematically. Transcripts were reviewed to develop a code list of the topics related to the research questions. Then, codes were applied manually. Text pertaining to the codes was organized in a matrix and translated into English. Finally, codes were classified into main categories and subcategories on the basis of differences and similarities. No software was used to analyze the data. In addition, the study also adhered to the RATS guidelines (relevance of study question, soundness of interpretive approach, appropriateness of qualitative method, and transparency of procedures) for reporting qualitative studies.

### Ethical considerations

Ethical clearance was obtained from institutional review board of Wollega University. Permission letter was also taken from the Benishangul Gumuz Regional State Health Bureau. Then, verbal informed consent was taken from each participant. The anonymity and confidentiality of the respondents were maintained.

## Result and discussions

### Profile of respondents

In the study, a total of 72 participants were included. Among these, the majority (n = 41) of them were caregivers. Four health extension workers, nineteen volunteer community health workers, three traditional healers, three private clinic and two drug vendor owners were also participated.

Of the participants, most (n = 60) of them were females. The majority of caregivers could not read and write and found in the age range between 18 to 34 years. The traditional healers were above 70 years of age and could not read and write.

### Perceived causes of childhood diarrhea

The vast majority of the study participants perceived that childhood diarrhea is caused by inadequate personal hygiene and poor environmental sanitation. This clearly indicates that many of the caregivers had the correct understanding of the causes of childhood diarrhea. The finding is also in line with a qualitative study done in rural Kenya [10]. However, a few participants associated the occurrence of childhood diarrhea with eruption of milk teeth, sucking hot breast milk, exposure to hot weather and feeding more breast milk. A 30 years old & illiterate mother elaborated how exposure to hot weather causes diarrhea as follows:

*“When children are exposed to hot weather, they may get diarrhea. For instance, since I am a farmer, I do the household chores including baking injera in the kitchen, carrying my child on my back. Therefore, if the child is exposed to hot weather, he/she gets diarrhea.”*

These findings clearly indicate the existence of knowledge gap on the etiology of childhood diarrhea. Thus, the study informs health officials and other stakeholders to develop a better strategy to avoid these types of misperceptions.

### Perception of childhood diarrhea as a common childhood disease

Many of the participants perceived that diarrhea is one of the three most common childhood diseases in the study area. The morbidity data report of the district health office (Assossa Woreda Health Office) and Benishangul Gumuz Regional State Health Bureau also indicated diarrhea was among the ten top diseases. In relation to this point, a 36th year old mother who completed 7th grade said the following during a focus group discussion:

*The common childhood diseases in our village are malaria, diarrhea and pneumonia. Diarrhea hurts a child and it might lead to death. If the diarrhea is bloody and a child does not get appropriate treatment soon, it can lead to death. In addition, a child with diarrhea faces loss of appetite and body weight. Therefore, diarrhea has severe effects on child growth.*

In contrast, a few of them indicated that diarrhea was not a major health problem of children. These participants argued that diarrhea should not be considered as a concern to child health and growth as most of the children got diarrhea which resolved without any severe health problems. This finding is consistent with a survey done in Pakistan [11]. Therefore, the study indicates the importance of making use of a number of different approaches to change the local misperceptions of the community towards childhood diarrhea.

The participants perceived that the occurrence of diarrhea is high during the rainy season mainly because of the contamination of water sources. Every year in February and March (the mango season), they perceived that many children got diarrhea because they picked plenty of mangoes that fell on the ground and ate them without cleaning. Thus, health education and hygiene promotion programs by HEW and non-governmental organizations (NGO) should be strengthened as the participants had already observed the impact in reducing diarrhea cases in the last few years.

### Homemade management of diarrhea

Most of the caregivers usually prepared homemade medications for mild cases of diarrhea, which they use as first aid before they get prescribed treatment at the nearby health facility. This homemade treatment is commonly practiced by the Amhara ethnic group. One of the health care professionals working in the private clinic explained it as follows:

*“The commonest homemade practice is giving boiled and cooled water with honey and Martriza (Tegera Birr) silver coin before bringing the sick children to us. Amazingly, the treatment works for them. They use this practice as a first aid,* i.e. *to save the lives of their children before bringing to the clinic as they do have a problem of getting transport on time to bring their children to town. The mothers give this traditional treatment as long as their child is able to take/drink it. As the treatment has a metallic taste, some of the children may not drink it.*

Generally, the practice of homemade treatments depends on the community’s perception of the causes of childhood diarrhea. For instance, if caregivers believe their children got diarrhea as a result of exposure to hot weather, they treat them using steam of leaf from boiled leaves of white ‘*Behar Zafe*’ (Eucalyptus), i.e. the caregivers boil the leaves and advise to inhale the steam. If the diarrhea does not stopped and the child does not show improvement, then the caregiver takes him/her to the nearby health facility.

These homemade treatments might lead children to severe complication of diarrhea including death as it does not provide the opportunity to get modern management by a health care provider [10]. So, advocating appropriate diarrheal management at the household level has paramount importance.

### Traditional management of childhood diarrhea

Traditional medications are commonly used by indigenous ethnic groups in the district. They are provided by traditional healers. Before prescribing medications, they ask the caregivers about the age of the child, duration and type of diarrhea. In addition, they also ask the sex of the child because the dose and duration of treatment vary. Then, they provide traditional medications called “*Sirsafe or Kebercho*” for watery, “*Bibi or Muqudjo*” for bloody, and “*Shekata*” for mucoid type of diarrhea (Table 1). All these medications are prepared from the roots of different trees. In addition, they also advise the caregivers about the time, dose and duration of providing the treatments.

**Table 1:** **Traditional medications (tree roots) provided by traditional healers for treating childhood diarrhea**

Type of diarrhea	Name of traditional medication	Dosage for children	Duration of the treatment	Remark
Watery diarrhea	*Sirsafe*	1 spoon a day	2–3 days	
Mucoid diarrhea	*Shekata*	Once in a day mixed with food	2–3 days	The medication is a sort of tablet and it is provided for children by mixing with food usually ‘*injera*’ and ‘*wot*’
Bloody diarrhea	*Bibi*	1 ½ spoon a day	2–5 days	If the diarrhea does not stop within 3 days, the medication can last for 5 days.

Therefore, interventions should also target traditional healers so as to achieve a positive impact on the burden of childhood diarrheal diseases.

### Communication media as source of information about childhood diarrhea

Almost all of the village residents do not have access to mass media like Radio, TV and newspaper. That is why they preferred live education sessions as a means of communication. This definitely creates a great challenge to provide health messages to the community. Thus, due attention should be given to reach the community.

### Limitations of the study

The process of selection of the participants may lead to bias. However, different approaches were used to validate the selected participant. In addition, these findings cannot be generalized to other areas rather it helps as baseline information for researchers and program planners.

## Conclusions

The majority of the participants perceived that childhood diarrhea is one of the major public health problems. Most of the caregivers had the correct understanding of the causes of diarrhea, but still diverse misperceptions still existed. Local homemade management of diarrhea was commonly practiced. However, indigenous ethnic groups preferred traditional treatments for their children. Thus, appropriate diarrhea control and prevention programs should be developed by considering the local culture and resources.
